# Supplementary material for: Open-source data pipeline for street-view images: A case study on community mobility during COVID-19 pandemic
Source: PLoS One. 2024 May 10;19(5):e0303180. doi: 10.1371/journal.pone.0303180 (PMC11086835; doi:10.1371/journal.pone.0303180)
Supplement: S1 File — (PDF) [file pone.0303180.s002.pdf]

SI Information

**Dep. Variable:**	Two_Or_More_Peds_per_Image	**R-squared:**	0.114
**Model:**	OLS	**Adj. R-squared:**	0.112
**Method:**	Least Squares	**F-statistic:**	40.82
**No. Observations:**	3171	**Prob (F-statistic):**	1.97e-76
**Df Residuals:**	3160	**Log-Likelihood:**	438.90
**Df Model:**	10		
**Covariance Type:**	nonrobust		

	**coef**	**std err**	**t**	**P> |t|**	**[0.025**	**0.975]**
**Intercept**	0.2590	0.016	16.091	0.000	0.227	0.291
**Spring**	0.0122	0.011	1.073	0.283	-0.010	0.034
**Summer**	0.0619	0.012	5.278	0.000	0.039	0.085
**Winter**	-0.0044	0.012	-0.352	0.725	-0.029	0.020
**Vaccine Available**	-0.0143	0.008	-1.883	0.060	-0.029	0.001
**Weekend**	-0.0264	0.011	-2.366	0.018	-0.048	-0.005
**Income Bracket 2**	-0.0981	0.017	-5.675	0.000	-0.132	-0.064
**Income Bracket 3**	-0.2080	0.017	-12.377	0.000	-0.241	-0.175
**Income Bracket 4**	-0.2346	0.018	-12.758	0.000	-0.271	-0.199
**Income Bracket 5**	-0.3230	0.025	-13.081	0.000	-0.371	-0.275
**More than 55.5% White**	0.1564	0.012	13.494	0.000	0.134	0.179

Table 1. OLS Regression Results for Detections per Image for the detections subset sharing an image with at least 1 other.

**Dep. Variable:**	Three_Or_More_Peds_per_Image	**R-squared:**	0.098
**Model:**	OLS	**Adj. R-squared:**	0.095
**Method:**	Least Squares	**F-statistic:**	34.23
**No. Observations:**	3171	**Prob (F-statistic):**	6.49e-64
**Df Residuals:**	3160	**Log-Likelihood:**	1864.6
**Df Model:**	10		
**Covariance Type:**	nonrobust		

	**coef**	**std err**	**t**	**P> |t|**	**[0.025**	**0.975]**
**Intercept**	0.1292	0.010	12.581	0.000	0.109	0.149
**Spring**	0.0026	0.007	0.356	0.722	-0.012	0.017
**Summer**	0.0309	0.007	4.128	0.000	0.016	0.046
**Winter**	-0.0053	0.008	-0.671	0.502	-0.021	0.010
**Vaccine Available**	0.0047	0.005	0.975	0.330	-0.005	0.014
**Weekend**	-0.0110	0.007	-1.544	0.123	-0.025	0.003
**Income Bracket 2**	-0.0657	0.011	-5.961	0.000	-0.087	-0.044
**Income Bracket 3**	-0.1289	0.011	-12.025	0.000	-0.150	-0.108
**Income Bracket 4**	-0.1472	0.012	-12.544	0.000	-0.170	-0.124
**Income Bracket 5**	-0.1926	0.016	-12.225	0.000	-0.223	-0.162
**More than 55.5% White**	0.0910	0.007	12.308	0.000	0.077	0.106

Table 2. OLS Regression Results for Detections per Image for the detections subset sharing an image with at least 2 others.

**Dep. Variable:**	Four_Or_More_Peds_per_Image	**R-squared:**	0.077
**Model:**	OLS	**Adj. R-squared:**	0.074
**Method:**	Least Squares	**F-statistic:**	26.38
**No. Observations:**	3171	**Prob (F-statistic):**	9.11e-49
**Df Residuals:**	3160	**Log-Likelihood:**	3100.6
**Df Model:**	10		
**Covariance Type:**	nonrobust		

	**coef**	**std err**	**t**	**P> |t|**	**[0.025**	**0.975]**
**Intercept**	0.0709	0.007	10.198	0.000	0.057	0.085
**Spring**	0.0009	0.005	0.186	0.853	-0.009	0.011
**Summer**	0.0197	0.005	3.878	0.000	0.010	0.030
**Winter**	-0.0052	0.005	-0.972	0.331	-0.016	0.005
**Vaccine Available**	0.0073	0.003	2.224	0.026	0.001	0.014
**Weekend**	-0.0041	0.005	-0.842	0.400	-0.014	0.005
**Income Bracket 2**	-0.0432	0.007	-5.787	0.000	-0.058	-0.029
**Income Bracket 3**	-0.0781	0.007	-10.764	0.000	-0.092	-0.064
**Income Bracket 4**	-0.0889	0.008	-11.186	0.000	-0.104	-0.073
**Income Bracket 5**	-0.1144	0.011	-10.721	0.000	-0.135	-0.093
**More than 55.5% White**	0.0527	0.005	10.522	0.000	0.043	0.063

Table 3. OLS Regression Results for Detections per Image for the detections subset sharing an image with at least 3 others.
